# Biomarkers in Exhaled Breath Condensate Are Not Predictive for Pulmonary Exacerbations in Children with Cystic Fibrosis: Results of a One-Year Observational Study

**DOI:** 10.1371/journal.pone.0152156

**Published:** 2016-04-06

**Authors:** Marieke van Horck, Ariel Alonso, Geertjan Wesseling, Karin de Winter—de Groot, Wim van Aalderen, Han Hendriks, Bjorn Winkens, Ger Rijkers, Quirijn Jöbsis, Edward Dompeling

**Affiliations:** 1 Department of Pediatric Pulmonology, School for Public Health and Primary Health Care (CAPHRI), Maastricht University Medical Centre (MUMC+), Maastricht, The Netherlands; 2 Leuven Biostatistics and Statistical Bioinformatics Centre, KU Leuven, Leuven, Belgium; 3 Department of Pulmonology, CAPHRI, MUMC+, Maastricht, The Netherlands; 4 Department of Pediatric Pulmonology, Wilhelmina Children’s Hospital, University Medical Centre Utrecht (UMCU), Utrecht, The Netherlands; 5 Department of Pediatric Respiratory Medicine, Emma Children’s Hospital, Academic Medical Centre (AMC), Amsterdam, The Netherlands; 6 Department of Pediatrics, Viecuri Medical Centre, Venlo, The Netherlands; 7 Department of Methodology and Statistics, CAPHRI, MUMC+, Maastricht, The Netherlands; 8 Laboratory of Medical Microbiology and Immunology, St Antonius Hospital, Nieuwegein, The Netherlands; 9 Department of Sciences, University College Roosevelt, Middelburg, The Netherlands; French National Centre for Scientific Research, FRANCE

## Abstract

**Background:**

Cystic Fibrosis (CF) is characterized by chronically inflamed airways, and inflammation even increases during pulmonary exacerbations. These adverse events have an important influence on the well-being, quality of life, and lung function of patients with CF. Prediction of exacerbations by inflammatory markers in exhaled breath condensate (EBC) combined with early treatment may prevent these pulmonary exacerbations and may improve the prognosis.

**Aim:**

To investigate the diagnostic accuracy of a set of inflammatory markers in EBC to predict pulmonary exacerbations in children with CF.

**Methods:**

In this one-year prospective observational study, 49 children with CF were included. During study visits with an interval of 2 months, a symptom questionnaire was completed, EBC was collected, and lung function measurements were performed. The acidity of EBC was measured directly after collection. Inflammatory markers interleukin (IL)-6, IL-8, tumor necrosis factor α (TNF-α), and macrophage migration inhibitory factor (MIF) were measured using high sensitivity bead based flow immunoassays. Pulmonary exacerbations were recorded during the study and were defined in two ways. The predictive power of inflammatory markers and the other covariates was assessed using conditionally specified models and a receiver operating characteristic curve (SAS version 9.2). In addition, *k*-nearest neighbors (KNN) algorithm was applied (SAS version 9.2).

**Results:**

Sixty-five percent of the children had one or more exacerbations during the study. The conditionally specified models showed an overall correct prediction rate of 55%. The area under the curve (AUC) was equal to 0.62. The results obtained with the KNN algorithm were very similar.

**Conclusion:**

Although there is some evidence indicating that the predictors outperform random guessing, the general diagnostic accuracy of EBC acidity and the EBC inflammatory markers IL-6, IL-8, TNF-α and MIF is low. At present it is not possible to predict pulmonary exacerbations in children with CF with the chosen biomarkers and the method of EBC analysis. The biochemical measurements of EBC markers should be improved and other techniques should be considered.

## Introduction

Cystic fibrosis (CF) is the most common life-shortening genetic disease in the Caucasian population, caused by a mutation in the cystic fibrosis transmembrane conductance regulator (CFTR) gene [1]. There are different types of mutations in this gene, all of them leading to a defective or absent CFTR protein [2]. The CFTR protein is expressed in the apical membrane of epithelial cells, and a defective protein causes problems in organs with a secretory function such as the lungs, intestines and pancreas. Hence CF is a multi-system disease. Nevertheless the course of this progressive disease is predominantly determined by respiratory symptoms and complications [3]. Respiratory deteriorations caused by alteration of the fragile homeostasis between airway pathogens and local host defenses are termed pulmonary exacerbations which are common in CF [4]. Pulmonary exacerbations present clinically with a variety of symptoms such as increased cough, increased sputum production, increased dyspnea, decreased energy level and appetite, weight loss and decreases in lung function parameters [5]. Currently these adverse events are treated with antibiotics when apparent, it is not yet possible to predict them. This means there is often a delay in starting treatment. An earlier treatment may positively influence the course of an exacerbation and improve long term prognosis in CF. However, to start treatment timely, it is necessary to detect pulmonary exacerbations at a very early stage.

Chronic inflammation of the airways is a major characteristic of CF. The inflammatory response is excessive and dysregulated, furthermore it plays an important role in both chronic bacterial infections and pulmonary exacerbations [6]. The CF airway contains several pro-inflammatory mediators (like tumor necrosis factor alpha [TNF-α], interleukin [IL]-6 and IL-8 and counter-regulatory molecules like IL-10) [7]. Macrophage migration inhibitory factor (MIF) is another pro-inflammatory cytokine important in the regulation of both the innate and acquired immune responses [8]. The MIF polymorphism, associated with lower promotor activity, is associated with milder lung disease in F508del CF patients [9]. Previous research in children with CF showed that inflammatory markers can be measured in exhaled breath condensate (EBC) [10]. In contrast to bronchoalveolar lavage fluid, induced sputum and endobronchial biopsies, EBC is a completely non-invasive, safe and fast technique, not bearing any health risks, and also feasible in young children [11]. In an earlier cross-sectional study in 98 children with CF and healthy controls, we found that inflammatory markers in EBC were able to indicate presence, severity and stability of CF disease [12]. A pilot study of our group showed that IL-6, IL-8, IL-10, TNF-α, and MIF, were predictive for exacerbations in children with CF [13]. Therefore, we hypothesized that pulmonary exacerbations can be predicted at an early stage by assessing the inflammatory markers IL-6, IL-8, TNF-α and MIF in EBC. The aim of this study was to investigate the diagnostic accuracy of this set of inflammatory markers in EBC to predict pulmonary exacerbations in children with CF.

## Methods

### Study design and setting

For this one-year observational cohort study (clinicaltrial.gov NCT01241890), children with CF between 5 and 18 years were recruited from three CF centers in the Netherlands (Maastricht, Amsterdam and Utrecht). Families were approached for the study by one of the pediatric pulmonologists during regular hospital visits and received written and oral information. The Medical Ethical Committee of the Maastricht University Medical Centre approved this study. Informed consent was signed by all parents and by children aged 12 years and older.

In the Maastricht University Medical Centre enrolment started in December 2011 and follow-up ended in May 2013. In the University Medical Centre Utrecht, the first children were enrolled in January 2012 and follow-up ended in June 2013. Finally, enrolment of children in the Amsterdam Medical Centre started in March 2012 and follow-up ended in August 2013.

Study visits were scheduled every 2 months during one year. To lessen the burden of the study and avoid loss to follow-up, we combined study visits with regular hospital visits as much as possible.

### Patients

CF disease was defined as the presence of characteristic clinical features (persistent pulmonary symptoms, meconium ileus, failure to thrive, steatorrhea) in combination with an abnormal sweat test (chloride > 60mM/L) and/or two CF mutations [14].

Exclusion criteria were: 1) severe cardiac abnormalities; 2) mental disability; 3) no technically adequate performance of measurements; 4) on waiting list for lung transplantation; 5) children colonized with *Burkholderia Cepacia or Methicillin Resistant Staphylococcus Aureus*; 6) participation in an intervention trial.

### Study parameters

The occurrence of a pulmonary exacerbation was the primary outcome measure which was defined in two ways: first, according to the definition used in the EPIC trial [15]; and second, when the responsible pediatric pulmonologist started a course of therapeutic antibiotics (oral and/or intravenous) considering the clinical symptoms as an expression of a pulmonary exacerbation.

The presence of an exacerbation according to the EPIC trial was established by one of the major criteria alone, or two of the minor signs, and fulfillment of symptom duration (duration of sign/symptoms ≥5 days or significant symptom severity) (Table 1).

**Table 1:** Definition of pulmonary exacerbation according to EPIC trial.

**Major criteria**
Decrease in FEV_1_ >10% from best baseline within past 6 months, unresponsive to beta-2 agonist
Oxygen saturation <90% on room air or >5% decline from previous baseline
New lobar infiltrate(s) or atelectasi(e)s on chest radiograph
Haemoptysis (more than streaks on more than one occasion in past week)
**Minor criteria**
Increased work of breathing or respiratory rate
New or increased adventitial sounds on lung exam
Weight loss >5% of body weight or decrease across 1 major percentile in weight percentile for age in past 6 months
Increased cough
Decreased exercise tolerance or level of activity
Increased chest congestion or change in sputum

Treatment of pulmonary exacerbations during the study occurred in accordance with the Dutch Central Guidance Committee (CBO) guideline [16], which closely resembles European [14] and American CF guidelines [17]

During every study visit, the same measurements took place: first, children completed a questionnaire, thereafter EBC collection took place, and finally, lung function measurements were performed. All measurements were carried out by extensively trained members of the research teams. The measurements were the same for all children.

#### Questionnaire

A questionnaire derived from the validated Dutch version of the revised Cystic Fibrosis Questionnaire (CFQ-R) was used to evaluate symptoms [18]. At 0, 6 and 12 months the CFQ-R was completed entirely by all children. The CFQ-R consists of 35–50 items divided into 7–9 domains (depending on age): physical functioning, energy and well-being, emotional state, social limitations, role limitations, body image, eating disturbances, treatment burden, and embarrassment. Furthermore, overall health perception and three symptom scales are included: respiratory, digestive and weight. Items require either a frequency response on a 4-point scale (‘all the time’ to ‘never’), a difficulty rating on a 4-point scale (‘a lot of difficulty’ to ‘no difficulty’), a true–false rating on a 4-point scale, or the selection of a statement that describes the patient (on a 3- or 4-point scale). The scores range from 0 to 100 with higher scores corresponding to higher quality of life.

#### Exhaled Breath Condensate (EBC)

Children breathed tidally for ten minutes, while wearing a nose-clip, through a mouthpiece connected to a two-way non-rebreathing valve (Hans Rudolph Inc, series 1420, Kansas City, USA) into a condenser system like described previously [19]. The two-way valve and swan-neck tubing, served as a saliva trap to the condenser system. When the child ceased the procedure, EBC was collected by pushing the plunger downwards and sampling in a collection tube. The glass condenser was cooled using a counter-current circulating ice-water pump. The cooling temperature was pre-set at 0.7°C. EBC acidity was determined without deaeration immediately after collection (handheld pH-meter, type PH1000H, and mic-microS7 pH sensor, VWR International B.V., NL, Germany). Condensate samples were snap-frozen using dry ice and stored at -80 degrees Celsius. Freeze-thaw cycles between collection and chemical analysis were avoided. To determine the levels of IL-6, IL-8, TNF-α, and MIF a commercially available high sensitivity bead-based flow immunoassays were used and concentrations were calculated using BioPlex software version 5.1 (Millipore, St Charles, MO, USA). All multiplex immunoassays were performed in 96 well format 1.2 μm filter bottom plates (Millipore, Amsterdam, The Netherlands) and a 12 point standard curve in duplicate was included on every plate. This standard curve consisted of the calibration fluids including two sequential dilutions to decrease the lower limit of quantification (LLoQ) of the assay. The median LLoQ’s assessed during analysis of the EBC samples were for IL-6: 114 fg/ml, IL-8: 25 fg/ml, TNF-α: 107 fg/ml and MIF 3710 fg/ml. In order to minimize interassay variation, positive and negative control samples were included. When fluorescence indices were below LLoQ, but above background, concentrations of samples were calculated by extrapolation. If concentrations could not be extrapolated, a concentration of 50% of the lowest measured concentration for the specific marker was imputed like described previously [20].

#### Lung function

The Masterscreen Pneumo (Carefusion, Houten, The Netherlands) was used to measure dynamic lung function parameters, according to ATS/ERS standards [21]. The highest values of three technically correct performed maneuvers were used for analysis. Recorded parameters were: Forced Expiratory Volume in 1 second (FEV_1_), forced vital capacity (FVC) and maximum expiratory flow at 50% of FVC (MEF_50_), all expressed as a percentage of the predicted normal value. Static lung function indices (total lung capacity [TLC], residual volume [RV], functional residual capacity [FRC], expiratory reserve volume [ERV] and intrathoracic gas volume [ITGV]) were determined at 0 and 12 months by means of body plethysmography using the Masterscreen Body (Carefusion, Houten, The Netherlands).

#### Covariates

Sex, Age, colonization with *Pseudomonas Aeruginosa* at inclusion, the use of prophylactic or therapeutic antibiotics, the use of corticosteroids, the time between visits and exacerbation at previous visit were considered as covariates. Information about the use of medication was checked at every study visit.

### Sample size

Forty-nine children were included in this observational cohort study. Based on an assumed prevalence of an exacerbation of 50% and assuming the worst case sensitivity and specificity of 0.50, the width of the confidence interval for sensitivity and specificity would be 0.4 (0.3–0.7).

### Statistical data analysis

For the statistical data analysis, SAS software package version 9.2 was used. To assess the prediction capacity of the biomarkers, a form of conditionally specified models, the so-called transition models were used. In transition models a measurement in a longitudinal sequence is described as a function of previous outcomes and covariates [22]. In this study the probability of a pulmonary exacerbation between the current and next visit was assessed using 2 conditional models. The first model (Model 1) included the biomarkers (IL-6, IL-8, TNF-α and MIF), pH of the EBC, lung function parameters (FEV1, FEV1% of predicted value, FVC and FVC % of predicted value), the time between visits and the presence of exacerbation at previous visit as predictors. The second model (Model 2) included all the predictors of the first model and age, sex, colonization with *Pseudomonas Aeruginosa* at inclusion, ABPA at inclusion, use of prophylactic antibiotics and use of inhalation corticosteroids. The data set was divided into a training and validation data sets. The training data set, containing the information of 36 randomly selected children, was used to estimate the predictive models and the predictive performance of the models was evaluated using the information in de validation data set containing the information of the remaining 13 children. The predictive capability of the models was primarily evaluated using the area under the corresponding ROC-curves. Additionally, the percentage of correct predictions was assessed using the validation data set.

Besides, the KNN algorithm was used to evaluate the predictive performance of the biomarkers in the validation data set using the information in the training data set. KNN is a non-parametric lazy learning algorithm [23]. The training samples are vectors of predictors or covariates, each with a class label. The training phase of the algorithm consists only of storing the feature vectors and class labels of the training samples.

All 49 patients were included in the statistical analysis, despite loss to follow-up or exclusion during the study. The proportion of missing values was generally low (less than 3.5%). The exception was pH that had about 7.6% of missing values. Only the presence of missing values in pH was considered for the construction of the predictive model, these missing values had no predictive value.

## Results

### Patients

**Fig 1:**
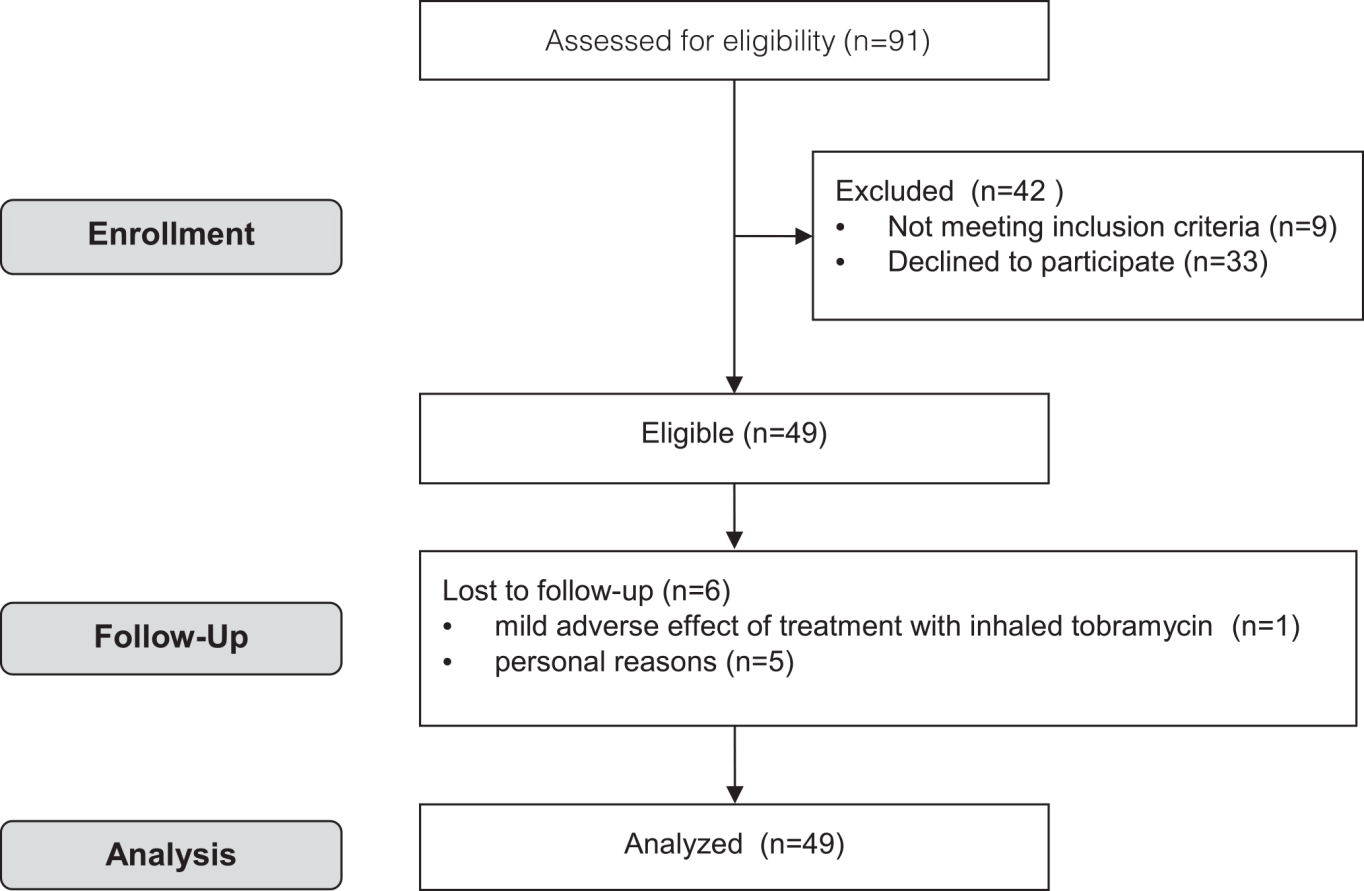
Consort flow diagram of the study.

Forty-nine children with CF were included in the study (Fig 1). One child was excluded after 10 months because of becoming a carrier of *Methicillin Resistant Staphylococcus Aureus*. Six out of the 49 children were lost to follow-up, mostly because of personal reasons (n = 5), or because of a mild adverse effect of inhaled tobramycin (n = 1). Data of the children who were lost to follow-up were included in the analysis (‘intention to treat’).

The mean age of the children was 10.3 years, more boys participated (63.3%) and the majority of the children had a homozygous DF508 mutation (73.5%). The lung function was good with a mean FEV_1_ of 87.4% of predicted value. The nutritional status was good as reflected by the BMI and BMI-SDS (Table 2). All children were included in the statistical analysis. Eighty-four percent of the children were evaluated seven times, 2 of the drop-outs had only 1 measurement.

**Table 2:** Baseline characteristics.

Characteristic	Total (n = 49)
Age, mean (SD)	10.3 (3.6)
Male sex, N (%)	31 (63.3)
Homozygous F508del, N (%)	36 (73.5)
*Pseudomonas Aeruginosa* at inclusion*, N (%)	15 (30.6)
Allergic Bronchopulmonary Aspergillosis (ABPA) at inclusion, N (%)	2 (4.0)
Pulmonary Exacerbations in 2 years before inclusion, N (%)	23 (46.9)
BMI, median (IQR)	16.8 (16.0–18.1)
BMI-SDS, mean (SD)	0.14 (0.83)
FEV_1_% predicted value, mean (SD)	87.4 (18.1)
FEV_1_/FVC, mean (SD)	0.80 (0.1)
TLC % predicted value, mean (SD) (n = 38)	101.1 (12.0)
RV % predicted value, mean (SD) (n = 37)	130.9 (42.7)
Prophylactic antibiotics, N (%)	28 (57.1)
Inhalation corticosteroids, N (%)	16 (32.7)

BMI, body mass index; FEV_1_, Forced Expiratory Volume in 1 second; FVC, Forced Vital Capacity; RV, Residual Volume; TLC, Total Lung Capacity.

* treated because of presence in sputum.

### Pulmonary exacerbations

When the EPIC trial definition for a pulmonary exacerbation was used, 32 children (65%) had 1 or more exacerbations during the study (Table 3). The prescription of therapeutic antibiotics by the pediatrician increased the percentage of children with exacerbation(s) to 88%.

**Table 3:** Number and percentages of children with pulmonary exacerbations (EPIC trial definition) during study.

Number of pulmonary exacerbations	Frequency	Percentage
0	17	34.7
1	12	24.5
2	8	16.3
3	6	12.2
4	3	6.1
5	3	6.1

### Description of biomarkers in EBC

There was a great variability in concentrations of measured biomarkers in EBC. The distribution of EBC acidity, and concentration of EBC biomarkers is given in Table 4.

**Table 4:** Acidity of EBC and concentrations of biomarkers in EBC.

Variable	Minimum	Maximum	Median	Mean	SD
pH	0	7.2	6.0	5.95	0.59
IL-6 (fg/ml)	0.35	107.0	0.35	3.25	10.08
IL-8 (fg/ml)	0.45	4710.80	0.45	24.09	272.16
TNF-α (fg/ml)	1.90	132.60	1.90	13.38	25.06
MIF (fg/ml)	92.53	291391.08	344.67	3141.64	17138.79

IL-6, interleukin-6; IL-8, interleukin-8; MIF, macrophage migration inhibitory factor; TNF-α, tumor necrosis factor α

### Diagnostic accuracy of biomarkers in EBC

The best predictive results were obtained using the most complex Model 2. The estimated parameters for this model are provided in S1 Table. This transition model correctly predicted 55% of the events (exacerbations or no exacerbations) in the validation dataset (Table 5). The prediction of an exacerbation was based on the probability of an exacerbation obtained from the prediction model with the probability > 0.5 as a cut-off point.

**Table 5:** Translational model prediction of pulmonary exacerbation (EPIC trial definition).

Pulmonary exacerbation	Prediction		
	No, n (%)	Yes, n (%)	Total, n (%)
**No**	39 (44)	8 (9)	47 (53)
**Yes**	32 (36)	10 (11)	42 (47)
**Total**	71 (80)	18 (20)	89 (100)

**Fig 2:**
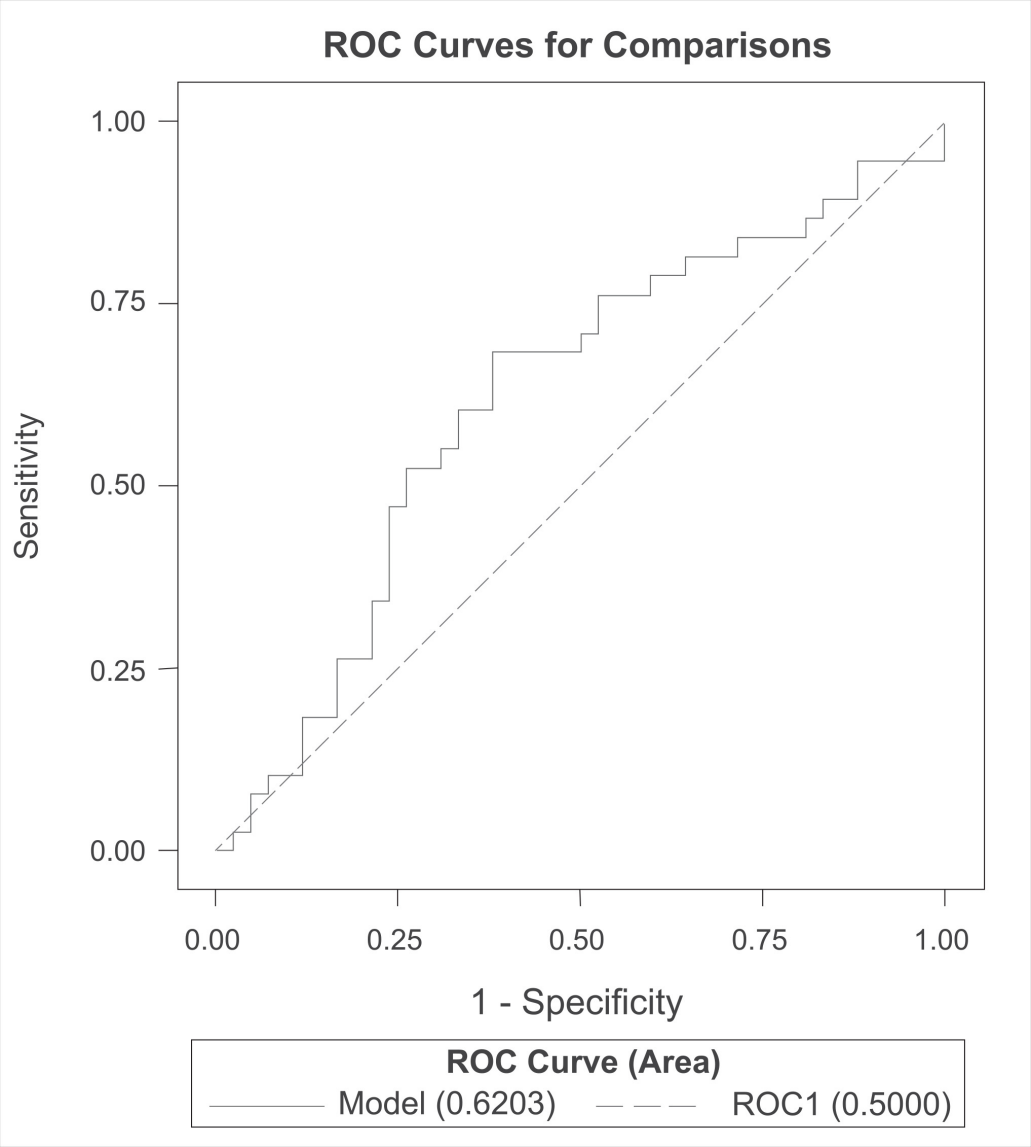
ROC-curve of predictive model of pulmonary exacerbations (EPIC trial definition).

However, the analysis of the ROC curve showed that the best predictions were obtained using a threshold of about 0.48 for the predictive probability of exacerbation, which led to a sensitivity of 0.70 and a specificity of 0.50. Thus, in the best scenario, the model would detect 70% of the exacerbations but would fail to predict absence of an exacerbation in 50% of the time. The predictive model (with threshold 0.48) led to an area under the curve of 0.62 (CI 0.49–0.75) (Fig 2).

The results obtained when the KNN algorithm was used were very similar. The KNN algorithm correctly predicted 59% of the events. If the second definition of a pulmonary exacerbation (when the responsible pediatric pulmonologist started a course of therapeutic antibiotics) was used, the overall correct prediction of the transitional model was 49% and the KNN algorithm predicted correctly 54% of the events.

## Discussion

In this study, we assessed the diagnostic accuracy of a set of biomarkers in EBC to predict pulmonary exacerbations in children with CF. Overall, we found low predictive power of the EBC acidity and the inflammatory markers IL-6, IL-8, TNFα and MIF. Neither the definition of exacerbations (the EPIC trial definition versus the prescription of therapeutic antibiotics by the pediatrician) nor the statistical method (transition models versus KNN algorithm) did significantly affect the results. At present, it is not possible to predict pulmonary exacerbations in children with CF by means of the chosen biomarkers and methods.

To our knowledge, only Horak et al performed a longitudinal study to investigate if an inflammatory marker, EBC nitrite, was helpful in monitoring lung disease in children with CF. They found that EBC nitrite could not predict pulmonary exacerbations or changes in pulmonary function or clinical and radiological scores [24]. Others have assessed inflammatory markers in EBC before and after treatment of pulmonary exacerbations. Bodini et al found that IL-8 EBC levels decreased and EBC acidity increased after antibiotic treatment of a pulmonary exacerbation [25]. In the study of Colombo et al., multiple biomarkers including IL-6, IL-8 and TNFα were measured in EBC before, during and after an acute exacerbation in adult CF patients [26]. Although IL-8 was positively correlated with CRP at the start and after 15 days of treatment, in a multivariate regression analysis, no significant associations between biomarkers in EBC and clinical variables were found [26]. Ojoo et al reported that EBC acidity was significantly lower during an exacerbation compared to stable disease [27]. In addition, inflammatory markers have been studied in induced sputum. Liou et al. reported that high mobility group box-1 protein (HMGB-1), a novel inflammatory cytokine, predicted time-to first acute pulmonary exacerbation and number of future exacerbations within 5 years [28]. In summary, biomarkers in EBC and induced sputum have been studied but results are variable and not yet applicable to clinical practice.

An important strength of our study is its prospective and longitudinal character: we have followed 49 children with CF during one year. We obtained EBC every 2 months, and recorded all exacerbations to investigate the predictive power of inflammatory markers in EBC. Furthermore, we used advanced statistical methods, transitional models and KNN algorithm, which take recurrent and dependent findings into account, and in this way fits the longitudinal nature of our study. This in contrast to other predictive models that presume one condition or finding independently leads to one outcome. Another strength is the use of two definitions of a pulmonary exacerbation as primary outcome measure, which minimizes information bias as a result of misclassification. If we had only used the stringent definition of the EPIC trial, we might have missed mild exacerbations, and incorrectly classified children as being stable when they were not. However, the use of two definitions accounts for heterogeneity of this primary outcome measure.

There are several explanations that could account for not being able to predict pulmonary exacerbations with the chosen biomarkers in EBC. First, in comparison with earlier studies [12, 19, 20], we had to switch to commercially available hypersensitive kits to analyze the EBC samples because the assay we used before was no longer available. The detection of cytokines and chemokines in EBC using this kit was lower than previously reported. Second, an explanation for the generally low concentrations of biomarkers in EBC may be the dilution of EBC as a medium. There are methods to correct for this dilution but each has specific disadvantages and therefore it is not recommended [11]. Third, the results of the biochemical analysis showed a large variability in concentrations of the measured biomarkers. This occurred in children with exacerbations as well as in children without exacerbations. Possible explanations for this variability of concentrations could be the used assays are not developed or adjusted for EBC, and the antibodies lack sensitivity and specificity [29]. In addition to the described factors influencing the biochemical analysis of the EBC samples, other aspects could have influenced our findings. The time frame between study visits might have been too long to detect relevant changes in biomarkers. In case we had collected EBC more frequently, it might have been easier to detect (small) changes in inflammatory markers. Besides, the interval between sampling of EBC and the start of a pulmonary exacerbation would have been shorter, which might have increased the chance to find an association between the increase in inflammation and the exacerbation. However, Sagel et al. reported biomarkers of inflammation to change over months rather than over days or weeks [30]. Which is in line with the time for clinical decision making whether or not to treat pulmonary exacerbations. Additionally in a comparable study in 40 children with asthma, we found that EBC acidity and IL-5 assessed every 2 months were significantly related to the exacerbation rate [31]. The choice of the biomarkers in the present study was based on the pathogenesis of CF, the possibility to measure the markers in EBC and on previous findings of predictive ability of exacerbations in a pilot study of our group [13]. Finally, our sample size was rather small, nonetheless, a larger group would probably not have improved our findings since not even a predictive trend was visible.

Although the collection of EBC is non-invasive, safe, fast and EBC originates directly from the (in this case chronically inflamed) airways, inflammatory markers in EBC currently do not contribute to the prediction of pulmonary exacerbations in children with CF. Future research should focus on development of EBC-specific sensitive assays for analysis of inflammatory markers in EBC that can cope with the strong dilution of EBC and the possible matrix effects. Furthermore, the potential of other techniques to analyze EBC like Nuclear Magnetic Resonance (NMR) spectroscopy, metabolomic profiling, gene expression or microbiome analyses should be explored.

In conclusion, we found that 2-monthly assessed inflammatory markers (IL-6, IL-8, TNFα and MIF) in EBC and acidity of EBC were not able to predict pulmonary exacerbations in children with CF. This may well be due to methodological problems concerning the biochemical analysis of EBC. Considering chronic airway inflammation is a major hallmark of CF and pulmonary exacerbations negatively influence the prognosis, it would be a big step forward to be able to measure this airway inflammation (directly and non-invasively) and predict upcoming exacerbations.

## Supporting Information

S1 Table Performance of acidity of EBC, inflammatory markers in EBC and covariates in prediction of pulmonary exacerbations. (DOCX)

## References

[1] O'Sullivan BP, Freedman SD. Cystic fibrosis. Lancet. 2009;373(9678):1891–904. 10.1016/S0140-6736(09)60327-5 19403164

[2] Ratjen F. Recent advances in cystic fibrosis. Paediatr Respir Rev. 2008;9(2):144–8. 10.1016/j.prrv.2008.01.004 18513677

[3] Zemanick ET, Harris JK, Conway S, Konstan MW, Marshall B, Quittner AL, et al Measuring and improving respiratory outcomes in cystic fibrosis lung disease: opportunities and challenges to therapy. J Cyst Fibros. 2010;9(1):1–16. 10.1016/j.jcf.2009.09.003 19833563 PMC2830746

[4] Rosenfeld M, Emerson J, Williams-Warren J, Pepe M, Smith A, Montgomery AB, et al Defining a pulmonary exacerbation in cystic fibrosis. J Pediatr. 2001;139(3):359–65. 11562614 10.1067/mpd.2001.117288

[5] Goss CH, Burns JL. Exacerbations in cystic fibrosis. 1: Epidemiology and pathogenesis. Thorax. 2007;62(4):360–7. 17387214 10.1136/thx.2006.060889 PMC2092469

[6] Chmiel JF, Berger M, Konstan MW. The role of inflammation in the pathophysiology of CF lung disease. Clin Rev Allergy Immunol. 2002;23(1):5–27. 12162106 10.1385/CRIAI:23:1:005

[7] Nichols DP, Chmiel JF. Inflammation and its genesis in cystic fibrosis. Pediatr Pulmonol. 2015;50 Suppl 40:S39–56. 10.1002/ppul.23242 26335954

[8] Cooke G, Armstrong ME, Donnelly SC. Macrophage migration inhibitory factor (MIF), enzymatic activity and the inflammatory response. Biofactors. 2009;35(2):165–8. 10.1002/biof.27 19322762

[9] Melotti P, Mafficini A, Lebecque P, Ortombina M, Leal T, Pintani E, et al Impact of MIF gene promoter polymorphism on F508del cystic fibrosis patients. PLoS One. 2014;9(12):e114274 10.1371/journal.pone.0114274 25503271 PMC4264759

[10] Bodini A, D'Orazio C, Peroni D, Corradi M, Folesani G, Baraldi E, et al Biomarkers of neutrophilic inflammation in exhaled air of cystic fibrosis children with bacterial airway infections. Pediatr Pulmonol. 2005;40(6):494–9. 16229003 10.1002/ppul.20336

[11] van Mastrigt E, de Jongste JC, Pijnenburg MW. The analysis of volatile organic compounds in exhaled breath and biomarkers in exhaled breath condensate in children—clinical tools or scientific toys? Clin Exp Allergy. 2015;45(7):1170–88. 10.1111/cea.12454 25394891

[12] Robroeks CM, Rosias PP, van Vliet D, Jobsis Q, Yntema JB, Brackel HJ, et al Biomarkers in exhaled breath condensate indicate presence and severity of cystic fibrosis in children. Pediatr Allergy Immunol. 2008;19(7):652–9. 10.1111/j.1399-3038.2007.00693.x 18312532

[13] Robroeks C, Jobsis Q, Braeckers R. Prediction of CF exacerbations by FeNO and non-invasive inflammatory markers in exhaled breath condensate. European Respiratory Society Congress; Berlin, Germany: European Respiratory Journal; 2008 p. 540s.

[14] Smyth AR, Bell SC, Bojcin S, Bryon M, Duff A, Flume P, et al European Cystic Fibrosis Society Standards of Care: Best Practice guidelines. J Cyst Fibros. 2014;13 Suppl 1:S23–42. 10.1016/j.jcf.2014.03.010 24856775

[15] Treggiari MM, Rosenfeld M, Mayer-Hamblett N, Retsch-Bogart G, Gibson RL, Williams J, et al Early anti-pseudomonal acquisition in young patients with cystic fibrosis: rationale and design of the EPIC clinical trial and observational study'. Contemp Clin Trials. 2009;30(3):256–68. 10.1016/j.cct.2009.01.003 19470318 PMC2783320

[16] CBO. Richtlijn diagnostiek en behandeling van cystic fibrosis. 2007.

[17] Flume PA, Mogayzel PJ Jr., Robinson KA, Goss CH, Rosenblatt RL, Kuhn RJ, et al Cystic fibrosis pulmonary guidelines: treatment of pulmonary exacerbations. Am J Respir Crit Care Med. 2009;180(9):802–8. 10.1164/rccm.200812-1845PP 19729669

[18] Klijn PH, van Stel HF, Quittner AL, van der Net J, Doeleman W, van der Schans CP, et al Validation of the Dutch cystic fibrosis questionnaire (CFQ) in adolescents and adults. J Cyst Fibros. 2004;3(1):29–36. 15463884 10.1016/j.jcf.2003.12.006

[19] Rosias PP, Robroeks CM, van de Kant KD, Rijkers GT, Zimmermann LJ, van Schayck CP, et al Feasibility of a new method to collect exhaled breath condensate in pre-school children. Pediatr Allergy Immunol. 2010;21(1 Pt 2):e235–44. 10.1111/j.1399-3038.2009.00909.x 19563465

[20] Rosias PP, Robroeks CM, Kester A, den Hartog GJ, Wodzig WK, Rijkers GT, et al Biomarker reproducibility in exhaled breath condensate collected with different condensers. Eur Respir J. 2008;31(5):934–42. 10.1183/09031936.00073207 18184682

[21] Miller MR, Hankinson J, Brusasco V, Burgos F, Casaburi R, Coates A, et al Standardisation of spirometry. Eur Respir J. 2005;26(2):319–38. 16055882 10.1183/09031936.05.00034805

[22] Molenberghs G VG. Models for Discrete Longitudinal Data. 1 ed: Springer-Verlag New York; 2005. 687 p.

[23] Hastie T TR, Friedman J. The Elements of Statistical Learning. corrected edition ed. New York: Springer; 2003.

[24] Horak F Jr., Moeller A, Singer F, Straub D, Holler B, Helbich TH, et al Longitudinal monitoring of pediatric cystic fibrosis lung disease using nitrite in exhaled breath condensate. Pediatr Pulmonol. 2007;42(12):1198–206. 17968999 10.1002/ppul.20719

[25] Bodini A, D'Orazio C, Peroni DG, Corradi M, Zerman L, Folesani G, et al IL-8 and pH values in exhaled condensate after antibiotics in cystic fibrosis children. Int J Immunopathol Pharmacol. 2007;20(3):467–72. 17880760 10.1177/039463200702000305

[26] Colombo C, Faelli N, Tirelli AS, Fortunato F, Biffi A, Claut L, et al Analysis of inflammatory and immune response biomarkers in sputum and exhaled breath condensate by a multi-parametric biochip array in cystic fibrosis. Int J Immunopathol Pharmacol. 2011;24(2):423–32. 21658316 10.1177/039463201102400215

[27] Ojoo JC, Mulrennan SA, Kastelik JA, Morice AH, Redington AE. Exhaled breath condensate pH and exhaled nitric oxide in allergic asthma and in cystic fibrosis. Thorax. 2005;60(1):22–6. 15618578 10.1136/thx.2003.017327 PMC1747154

[28] Liou TG, Adler FR, Keogh RH, Li Y, Jensen JL, Walsh W, et al Sputum biomarkers and the prediction of clinical outcomes in patients with cystic fibrosis. PLoS One. 2012;7(8):e42748 10.1371/journal.pone.0042748 22916155 PMC3416785

[29] van Vliet D, Alonso A, Rijkers G, Heynens J, Rosias P, Muris J, et al Prediction of asthma exacerbations in children by innovative exhaled inflammatory markers: results of a longitudinal study. PLoS One. 2015;10(3):e0119434 10.1371/journal.pone.0119434 25799487 PMC4370663

[30] Sagel SD, Chmiel JF, Konstan MW. Sputum biomarkers of inflammation in cystic fibrosis lung disease. Proc Am Thorac Soc. 2007;4(4):406–17. 17652508 10.1513/pats.200703-044BR PMC2647605

[31] Robroeks CM, van Vliet D, Jobsis Q, Braekers R, Rijkers GT, Wodzig WK, et al Prediction of asthma exacerbations in children: results of a one-year prospective study. Clin Exp Allergy. 2012;42(5):792–8. 10.1111/j.1365-2222.2012.03992.x 22515395

