# Supplementary material for: Biomarkers in Exhaled Breath Condensate Are Not Predictive for Pulmonary Exacerbations in Children with Cystic Fibrosis: Results of a One-Year Observational Study
Source: PLoS One. 2016 Apr 6;11(4):e0152156. doi: 10.1371/journal.pone.0152156 (PMC4822839; doi:10.1371/journal.pone.0152156)
Supplement: S1 Table — (DOCX) [file pone.0152156.s001.docx]

| **Inflammatory markers** | **Estimate** | **95% CI** | **p-value** |
| --- | --- | --- | --- |
| pH | 0.6471 | -0.3808, 1.6750 | 0.2172 |
| IL-6 | 0.0259 | -0.0036, 0.0553 | 0.0853 |
| IL-8 | -0.0097 | -0.0295, 0.0101 | 0.3377 |
| TNF-α | 0.0054 | -0.0167, 0.0274 | 0.6325 |
| MIF | -0.0001 | -0.0002, 0.0001 | 0.5690 |
| Age | -0.0360 | -0.3948, 0.3227 | 0.8440 |
| Gender | -0.0950 | -102780, 1.0879 | 0.8749 |
| *Pseudomonas Aeruginosa* at inclusion | -0.5893 | -1.7274, 0.5487 | 0.3101 |
| Use of prophylactic antibiotics | -0.0167 | -0.2305, 0.1972 | 0.8786 |
| Use of corticosteroids | -0.2569 | -0.8263, 0.3124 | 0.3764 |
| FEV_1_ % of predicted value | -0.0538 | -0.1682, 0.0605 | 0.3563 |
| FVC % of predicted value | -0.0001 | -0.1115, 0.1114 | 0.9992 |
| Time between visit and exacerbation at previous visit | -0.0097 | -0.1200, 0.1007 | 0.8635 |

IL-6, interleukin-6; IL-8, interleukin-8; MIF, macrophage migration inhibitory factor; TNF-α, tumor necrosis factor α; FEV_1_, Forced Expiratory Volume in 1 second; FVC, Forced Vital Capacity.
